# Baclofen mediates neuroprotection on hippocampal CA1 pyramidal cells through the regulation of autophagy under chronic cerebral hypoperfusion

**DOI:** 10.1038/srep14474

**Published:** 2015-09-28

**Authors:** Li Liu, Chang-jun Li, Yun Lu, Xian-gang Zong, Chao Luo, Jun Sun, Lian-jun Guo

**Affiliations:** 1 Department of Biochemistry and Molecular Biology, School of Basic Medicine, Tongji Medical College, Huazhong University of Science and Technology, Wuhan 430030, PR China; 2 Department of Pharmacology, School of Basic Medicine, Tongji Medical College, Huazhong University of Science and Technology, Wuhan 430030, PR China; 3 Key Laboratory of Drug Target Research and Pharmacodynamic Evaluation, Hubei Province, Wuhan 430030, China; 4 Neurology Department, Huanggang central hospital, Hubei Province, Huanggang, 438000, PR China; 5 Center for Integrated Protein Science (CIPSM) and Zentrum für Pharmaforschung, Department Pharmazie, Ludwig-Maximilians-Universität München, 80539 Munich, Germany

## Abstract

GABA receptors play an important role in ischemic brain injury. Studies have indicated that autophagy is closely related to neurodegenerative diseases. However, during chronic cerebral hypoperfusion, the changes of autophagy in the hippocampal CA1 area, the correlation between GABA receptors and autophagy, and their influences on hippocampal neuronal apoptosis have not been well established. Here, we found that chronic cerebral hypoperfusion resulted in rat hippocampal atrophy, neuronal apoptosis, enhancement and redistribution of autophagy, down-regulation of Bcl-2/Bax ratio, elevation of cleaved caspase-3 levels, reduction of surface expression of GABA_A_ receptor α1 subunit and an increase in surface and mitochondrial expression of connexin 43 (CX43) and CX36. Chronic administration of GABA_B_ receptors agonist baclofen significantly alleviated neuronal damage. Meanwhile, baclofen could up-regulate the ratio of Bcl-2/Bax and increase the activation of Akt, GSK-3β and ERK which suppressed cytodestructive autophagy. The study also provided evidence that baclofen could attenuate the decrease in surface expression of GABA_A_ receptor α1 subunit, and down-regulate surface and mitochondrial expression of CX43 and CX36, which might enhance protective autophagy. The current findings suggested that, under chronic cerebral hypoperfusion, the effects of GABA_B_ receptors activation on autophagy regulation could reverse neuronal damage.

Autophagy is an important catabolic process that eliminates damaged or unnecessary protein and organelle by delivering them to lysosomes for degradation^1^. Under physiological conditions, a low level of basal autophagy is crucial in the maintenance of normal intracellular homeostasis^2^, and is involved in various fundamental cellular processes such as protein and organelle quality control, development, differentiation and immunity^3^. Under stress conditions, autophagy activity at a moderate level seems to be essential for cell adaptation and survival^4^. However, excessive autophagy induced by nutrient starvation or stress may promote cell death^5^,^6^. More importantly, studies have reported that autophagy can play both beneficial and detrimental roles in many pathological conditions, such as cancer^7^ and cardiac ischemia^8^. In recent years, increasing evidence has indicated that autophagy in the central nervous system (CNS) is significantly enhanced under the conditions of hypoxia and ischemia. However, the role of autophagy in ischemic neurons remains controversial. Some reports have shown that autophagy plays an important role in protecting neurons from ischemia-induced death^4^,^9^,^10^. Inversely, many studies have demonstrated that autophagy in brain ischemia may contribute to neural damage, and inhibition of autophagy can attenuate cerebral ischemia-associated neuronal damage^11^,^12^,^13^,^14^,^15^,^16^,^17^. Thus, the survival or death contribution of autophagy in neuronal cells requires further study. Recently, evidence shows that autophagy is a process involved in neurodegenerative disorders such as Alzheimer’s disease, Parkinson’s disease and Huntington’s disease^18^,^19^,^20^,^21^,^22^,^23^,^24^,^25^,^26^,^27^,^28^. However, the role of autophagy activation in the chronic ischemic rats with possible features of vascular dementia and its potential applications in pharmacotherapy are still to be determined.

γ-aminobutyric acid (GABA) is the main inhibitory neurotransmitter and plays a crucial role in modulating the excitatory-inhibitory balance in the mammalian brain^29^. There are three main types of GABA receptors, the ionotropic GABA_A_ receptor and GABA_C_ receptor, and the metabotropic GABA_B_ receptor. GABA_A_ and GABA_B_ receptors have been shown to play a neuroprotective role in experimental models of middle cerebral artery occlusion (MCAO)^30^, transient brain ischemia^31^,^32^,^33^,^34^,^35^,^36^ and oxygen-glucose deprivation (OGD)^31^,^32^,^37^,^38^,^39^,^40^. Besides, GABA_B_ receptors can regulate the GABA_A_ receptors function in bullfrog DRG neurons^41^ and dentate gyrus granule cells^42^. However, little is known about the co-regulation of GABA_B_ receptors and GABA_A_ receptors and the influences of them on autophagy in the hippocampal CA1 area under chronic cerebral hypoperfusion.

Studies have shown that GABA_A_ receptors are involved in down-regulating the expression of Cx43^43^ and Cx36^44^ in the CNS. Recently, the effects of neuronal and glial gap-junctional communication (GJC) on neuroprotection became a noticeable focus. GJC is an important component in direct cell-to-cell communication that contributes to the maintenance of tissue homeostasis. Gap-junctional channels are formed by connexins. It has been reported that Cx26, Cx30, Cx32, Cx36 and Cx43 are expressed in the CNS^45^. In vertebrates, Cx43 is the most abundantly expressed connexins^46^ which is found only in astrocyte gap junctions, and Cx36 is only in neurons^47^,^48^. Recently, elevated CX43 and CX36 expression resulting from the neurological damage has been proposed^44^,^49^,^50^,^51^. More recently, Bejarano *et al.* have reported that plasma-membrane-localized Cx proteins constitutively down-regulate autophagy through a direct interaction with several autophagy-related proteins^52^. However, the potential roles of connexins in neuronal injury and autophagy under chronic cerebral hypoperfusion are poorly understood.

In the present study, we used a rat model of chronic cerebral hypoperfusion induced by permanent occlusion of bilateral common carotid arteries (2VO) to evaluate the changes of autophagy and the influences of them on neuronal apoptosis in the hippocampal CA1 area. We also investigated the possible mechanisms of correlation between GABA_B_ receptors, GABA_A_ receptors and autophagy in the hippocampal CA1 area of 2VO rats.

## Results

### Baclofen corrected excessive autophagy and decreased cleaved caspase-3 levels following OGD-Rep injury in brain slice models

**Figure 1:**
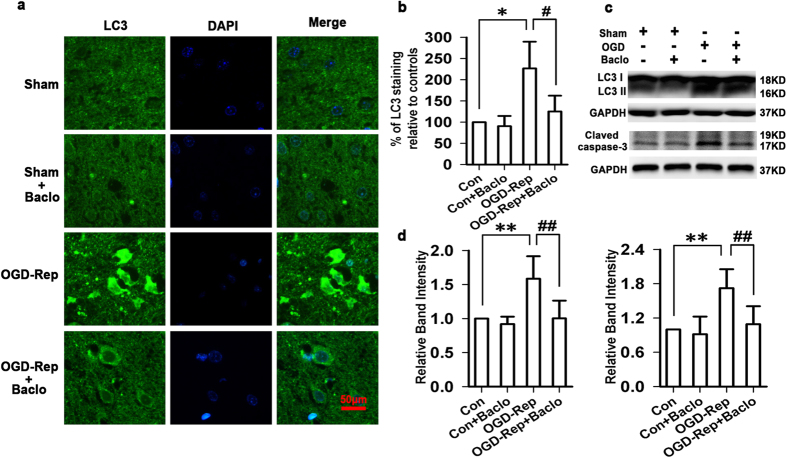
Baclofen corrected excessive autophagy and decreased cleaved caspase-3 levels following OGD-Rep injury in brain slice models. (**a**) Representative photomicrographs of immunohistochemical staining with anti-LC3 antibody in cortex (scale bar, 50 μm). In sham group, the LC3 immunoreactivity was very low. Treatment with baclofen in sham group had no significant change in the LC3 immunoreactivity. In the brain slice model of OGD-Rep injury, the LC3 immunoreactivity was robustly elevated compared with sham group, whereas in the OGD-Rep+baclofen (100 μM) group, the LC3 immunoreactivity was declined towards basal levels (n = 4 in each group). (**b**) Quantitative analysis of the LC3 immunoreactivity. (**c**,**d**) The protein levels of LC3 II were significantly increased in OGD-Rep group, but baclofen markedly alleviated excessive autophagy. OGD-Rep injury given rise to a significant increase in cleaved caspase-3 levels, baclofen significantly decreased cleaved caspase-3 levels (n = 4 in each group). ^*^P < 0.05 and ^**^P < 0.01 vs sham group; ^#^*P* < 0.05 and ^##^*P* < 0.01 vs OGD-Rep group.

In our preliminary experiments, we found that, in the brain slice model of OGD-Rep injury, the LC3 immunoreactivity was robustly elevated compared with sham group, whereas in the OGD-Rep+baclofen (100 μM) group, the LC3 immunoreactivity was declined towards basal levels. Treatment with baclofen in sham group had no significant change in the LC3 immunoreactivity (Fig. 1a,b). Correspondingly, the protein level of LC3-II was significantly increased in OGD-Rep group, but baclofen markedly alleviated excessive autophagy. Besides, our preliminary results also showed that OGD-Rep injury given rise to a significant increase in cleaved caspase-3 levels, and baclofen significantly decreased cleaved caspase-3 levels (Fig. 1c,d).

### Baclofen inhibited autophagy of hippocampal CA1 area under chronic cerebral hypoperfusion

**Figure 2:**
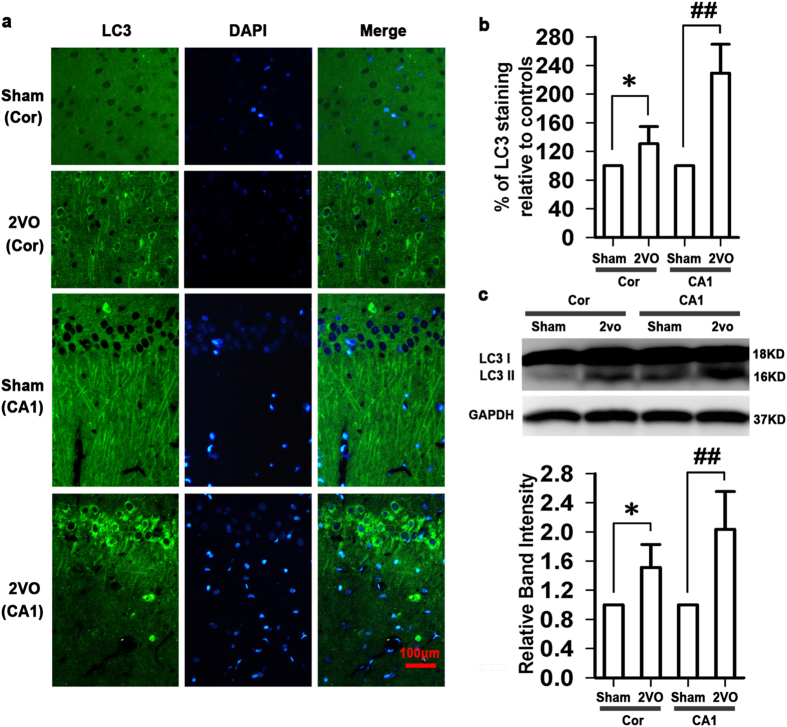
Activation of autophagy in cortex and hippocampal CA1 area under chronic cerebral hypoperfusion. (**a**) Representative photomicrographs of immunohistochemical staining with anti-LC3 antibody in cortex (scale bar, 100 μm). Five weeks after induction of hypoperfusion, the LC3 immunoreactivity was slightly but significantly increased in cortex, however, a robust increase in the LC3 immunoreactivity was observed in hippocampal CA1 area (n = 4 in each group). (**b**) Quantitative analysis of the LC3 immunoreactivity. (**c**) The protein expression of LC3 II in cortex and hippocampal CA1 area (n = 4 in each group). Blots shown have been cropped to fit space requirements and run under the same experimental conditions. *P < 0.05 vs sham-operated rats (cortex), ^##^*P* < 0.01 vs sham-operated rats (hippocampal CA1 area).

**Figure 3:**
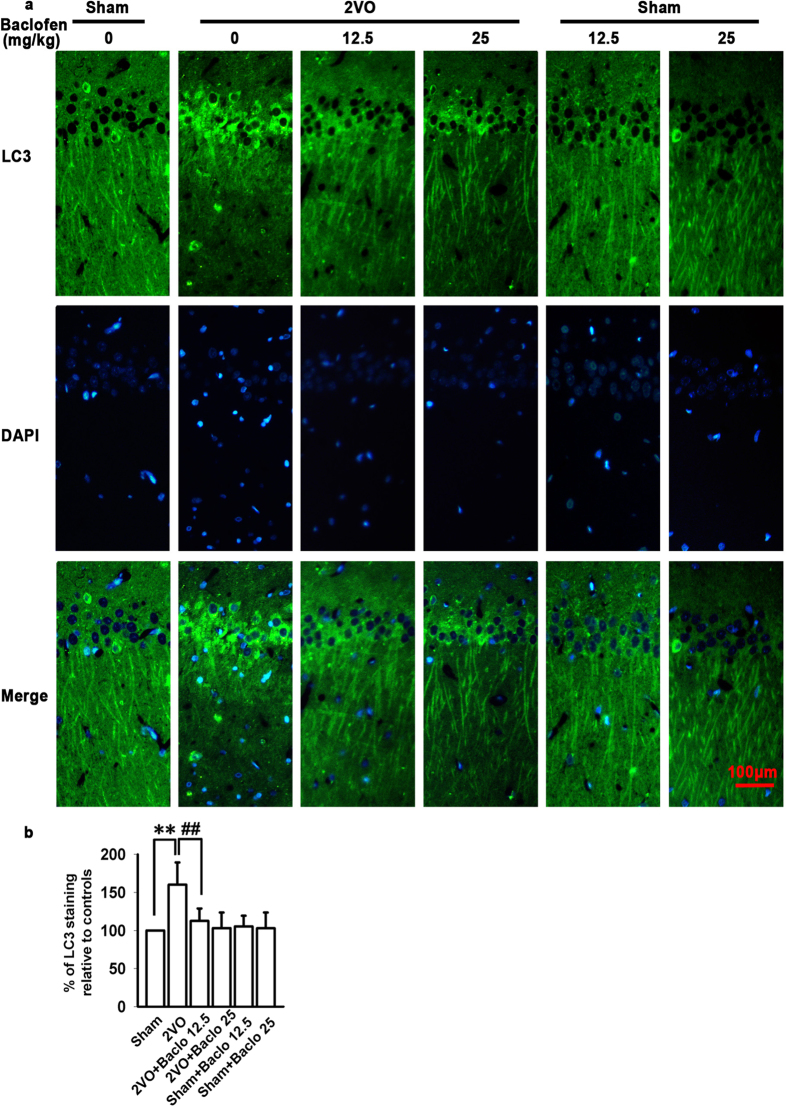
The role of baclofen in autophagy of hippocampal CA1 area under chronic cerebral hypoperfusion. (**a**) Representative photomicrographs of immunohistochemical staining with anti-LC3 antibody in hippocampal CA1 area (scale bar, 100 μm). In the sham-operated group, LC3 immunoreactivity was low and uniformly distributed along CA1 pyramidal cell axons, chronic cerebral hypoperfusion led to a distribution of LC3 immunoreactivity from CA1 pyramidal cell axons to abundant punctate structures in the cell body. Chronic treatment with baclofen significantly decreased the LC3 immunoreactivity and prevented LC3 redistribution. Treatment with baclofen at 12.5 mg/kg and 25 mg/kg in sham-operated rats had no significant effect on the LC3 immunoreactivity. (**b**) Quantitative analysis of the LC3 immunoreactivity (n = 4 in each group). ^**^P < 0.01 vs sham-operated rats; ^##^*P* < 0.01 vs 2VO rats.

**Figure 4:**
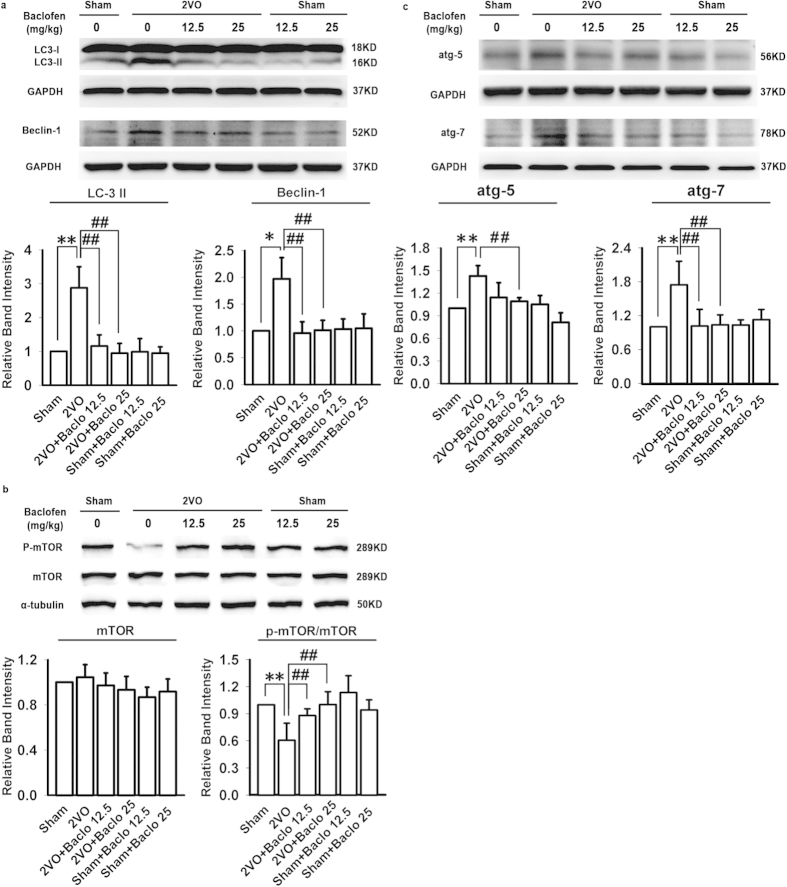
Baclofen reversed the changes of protein markers characteristic for autophagy in hippocampal CA1 area under chronic cerebral hypoperfusion. (**a**–**c**) Five weeks after induction of hypoperfusion, p-mTOR was significantly decreased, and LC3-II, Beclin 1, atg5 and atg7 were significantly increased, and baclofen could reverse the changes of these proteins expression. Treatment with baclofen at 12.5 mg/kg and 25 mg/kg in sham-operated rats did not change the expression of LC3-II, mTOR, p-mTOR, Beclin 1, atg5 and atg7 compared with sham-operated rats (n = 4 in each group). Blots shown have been cropped to fit space requirements and run under the same experimental conditions. ^*^P < 0.05 and ^**^P < 0.01 vs sham-operated rats; ^##^*P* < 0.01 vs 2VO rats.

To investigate whether autophagy is involved in the neuroprotection of GABA_B_ receptors activation under chronic cerebral hypoperfusion, we firstly examined the activation of autophagy in cortex and hippocampal CA1 area. As shown in Fig. 2, five weeks after induction of hypoperfusion, the LC3 immunoreactivity was slightly but significantly increased in cortex. However, in hippocampal CA1 area, we observed a robust increase in the LC3 immunoreactivity, which was consistent with the protein expression of LC3-II. Hippocampus is the area that displays the most characteristic neuropathological damage in neurodegenerative disorders and hippocampal CA1 area is one of the brain regions most sensitive to ischemia. Thus, in the follow-up experiments, we further investigated the role of baclofen in autophagy of hippocampal CA1 area under chronic cerebral hypoperfusion. Our results revealed that LC3 immunoreactivity was low in the sham-operated group, and uniformly distributed along CA1 pyramidal cell axons. Chronic cerebral hypoperfusion led to a redistribution of LC3 immunoreactivity from CA1 pyramidal cell axons to abundant punctate structures in the cell body. Chronic treatment with baclofen significantly decreased the LC3 immunoreactivity and prevented LC3 redistribution (Fig. 3a,b). To further confirm that baclofen could suppress chronic cerebral hypoperfusion-induced autophagy, we identified the expression of protein markers characteristic for autophagy, such as p-mTOR, Beclin 1, atg5, atg7 and LC3-II in the hippocampal CA1 area with Western blot analyses. Our results showed that, five weeks after induction of hypoperfusion, p-mTOR was significantly decreased, and LC3-II, Beclin 1, atg5 and atg7 were significantly increased. Baclofen could reverse the changes of these proteins expression. Treatment with baclofen at 12.5 mg/kg and 25 mg/kg in sham-operated rats did not change the expression of p-mTOR, LC3-II, Beclin 1, atg5 and atg7 compared with sham-operated rats (Fig. 4a,b).

### Baclofen diminished chronic hypoperfusion-induced neuronal apoptosis

**Figure 5:**
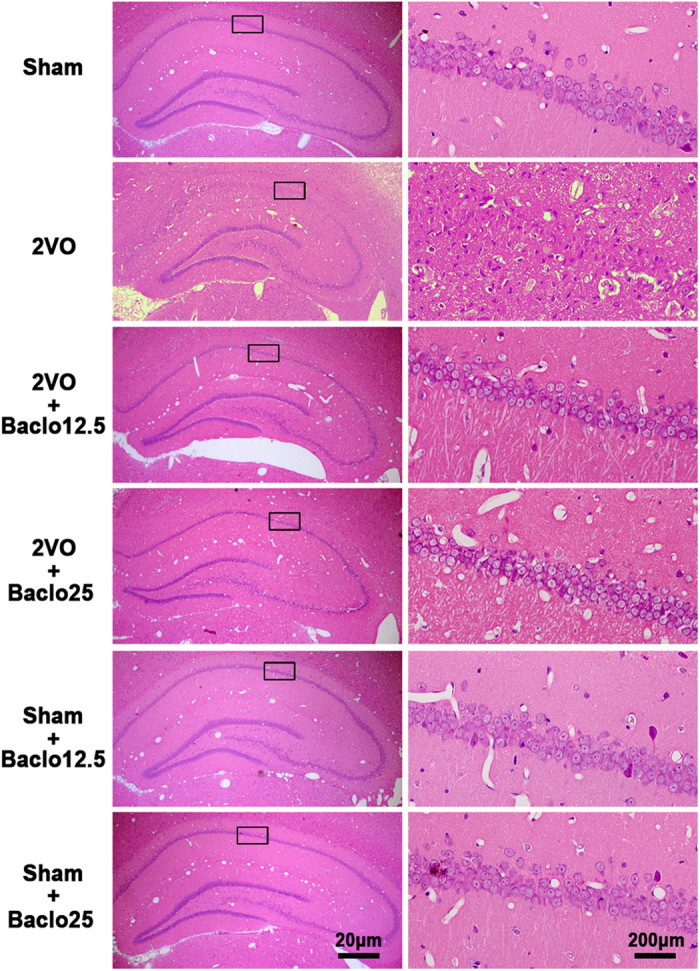
Hematoxylin and Eosin (H&E) staining. Example of H&E -strained sections of the hippocampus of each group (scale bar, 20 μm or 200 μm). Five weeks after induction of hypoperfusion, hippocampal atrophy and significant neuronal loss in hippocampal CA1 area were detected. Chronic treatment with baclofen markedly diminished hippocampal atrophy and hippocampal CA1 area neuronal loss. Treatment with baclofen at 12.5 mg/kg and 25 mg/kg in sham-operated rats had no significant effect on the morphology of CA1 pyramidal cells.

**Figure 6:**
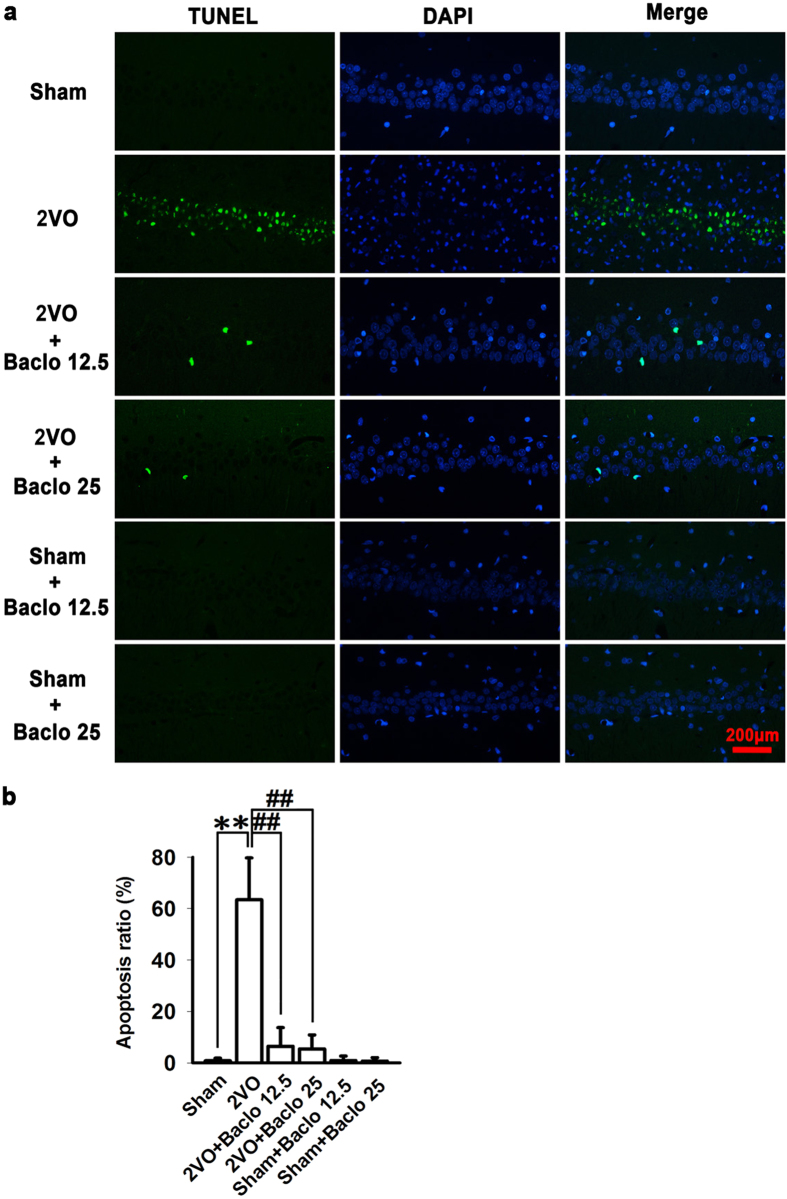
TUNEL staining of hippocampal CA1 area. (**a**) Representative photomicrographs of immunohistochemical staining with TUNEL (scale bar, 200 μm). TUNEL-stained positive cells were significantly increased in hippocampal CA1 area in 2VO rats. Treatment with baclofen at 12.5 mg/kg and 25 mg/kg in 2VO rats significantly reduced the numbers of TUNEL-stained positive cells. (**b**) Quantitative analysis of TUNEL staining. Apoptosis ratio = (number of TUNEL-positive nuclei/number of total nuclei) × 100% (n = 4 in each group). ^**^P < 0.01 vs sham-operated rats; ^##^*P* < 0.01 vs 2VO rats.

**Figure 7:**
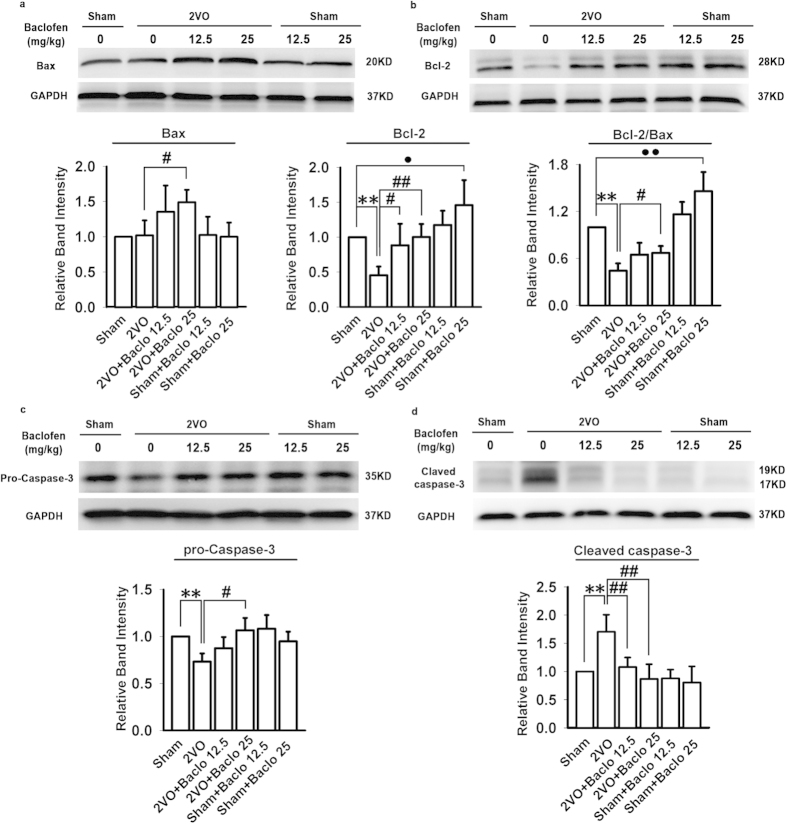
Baclofen attenuated chronic hypoperfusion-induced neuronal apoptosis. (**a**) Five weeks after induction of hypoperfusion, the expression of Bax was no significant change compared with sham-operated rats. Treatment with baclofen at 25 mg/kg in 2VO rats significantly enhanced the expression of Bax. Treatment with baclofen at 12.5 mg/kg and 25 mg/kg in sham-operated rats did not change the expression of Bax compared with sham-operated rats (n = 4 in each group). (**b**) Five weeks after induction of hypoperfusion, the expression of Bcl-2 was significantly decreased, treatment with baclofen at 12.5 mg/kg and 25 mg/kg in 2VO rats significantly increased the expression of Bcl-2. Treatment with baclofen at 12.5 mg/kg and 25 mg/kg in sham-operated rats significantly increased the expression of Bcl-2 compared with sham-operated rats (n = 4 in each group). Our results revealed that chronic cerebral hypoperfusion significantly decreased the ratio of Bcl-2/Bax in the hippocampal CA1 region, and baclofen could up-regulate Bcl-2/Bax ratio. (**c**) Five weeks after induction of hypoperfusion, the expression of pro-caspase-3 was significantly decreased compared with sham-operated rats, and treatment with baclofen recovered pro-caspase-3 expression (n = 4 in each group). (**d**) Five weeks after induction of hypoperfusion, the levels of cleaved caspase-3 in hippocampal CA1 cells were significantly increased, chronic treatment with baclofen significantly reduced cleaved caspase-3 levels (n = 4 in each group). Blots shown have been cropped to fit space requirements and run under the same experimental conditions. ^**^P < 0.01 vs sham-operated rats; ^#^*P* < 0.05 and ^##^*P* < 0.01 vs 2VO rats; ^•^*P* < 0.05 and ^••^*P* < 0.01 vs sham-operated rats.

In the present study, H&E and TUNEL staining was used to examine the influence of baclofen on degenerative changes of hippocampal CA1 area. Five weeks after induction of hypoperfusion, hippocampal atrophy and significant neuronal loss in hippocampal CA1 area were detected. Chronic treatment with baclofen markedly diminished hippocampal atrophy and neuronal loss in hippocampal CA1 area (Fig. 5). Besides, TUNEL-stained positive cells were significantly increased in hippocampal CA1 area in 2VO rats. Treatment with baclofen at 12.5 mg/kg and 25 mg/kg in 2VO rats significantly reduced the number of TUNEL-stained positive cells (Fig. 6a,b). Furthermore, we found that, five weeks after induction of hypoperfusion, the expression of Bax was not significantly changed compared with sham-operated rats. Treatment with baclofen at 25 mg/kg in 2VO rats significantly enhanced the expression of Bax. Treatment with baclofen at 12.5 mg/kg and 25 mg/kg in sham-operated rats did not change the expression of Bax compared with sham-operated rats. Besides, five weeks after induction of hypoperfusion, the expression of Bcl-2 was significantly decreased, treatment with baclofen recovered Bcl-2 expression. Treatment with baclofen at 12.5 mg mg/kg and 25 mg/kg in sham-operated rats significantly increased the expression of Bcl-2 compared with sham-operated rats. Our results revealed that chronic cerebral hypoperfusion significantly decreased the ratio of Bcl-2/Bax in the hippocampal CA1 region, and baclofen could up-regulate Bcl-2/Bax ratio (Fig. 7a,b). We also found that, five weeks after induction of hypoperfusion, the expression of pro-caspase-3 was significantly decreased compared with sham-operated rats, and treatment with baclofen recovered pro-caspase-3 expression (Fig. 7c). We further evaluated the levels of cleaved caspase-3 (an activated form of caspase-3) in each group. Our results showed that hypoperfusion resulted in a significant increase in cleaved caspase-3 levels of hippocampal CA1 cells, chronic treatment with baclofen significantly reduced cleaved caspase-3 levels (Fig. 7d).

### Baclofen could enhance the phosphorylation of protein kinase B (Akt) (Ser473), glycogen synthase kinase 3β (GSK-3β) (Ser-9) and extracellular regulated protein kinases 1/2 (ERK1/2)

**Figure 8:**
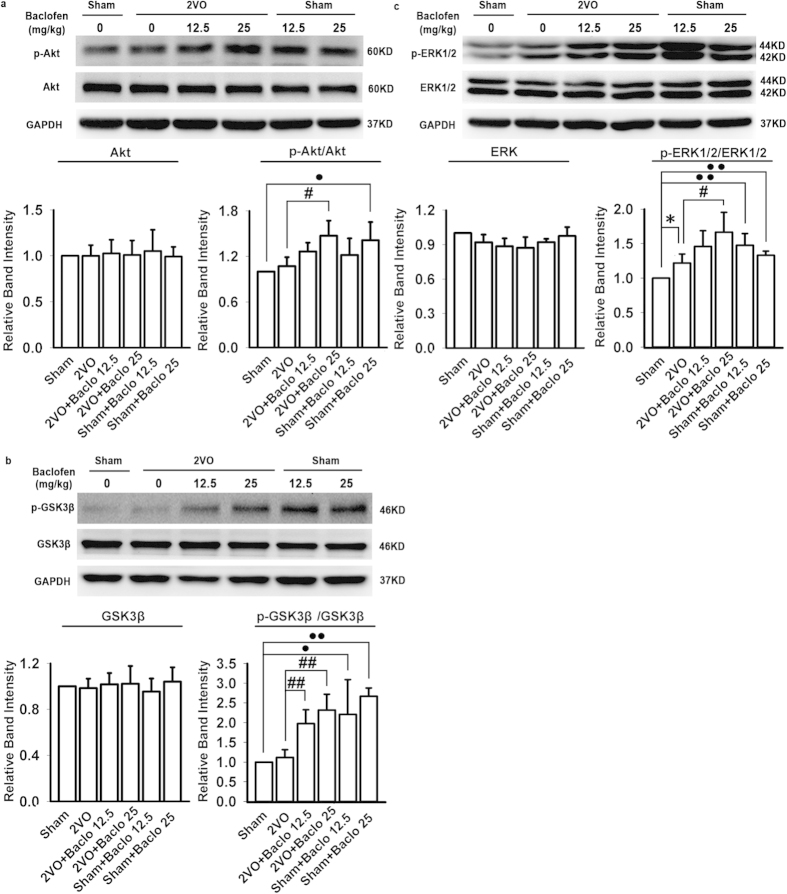
Baclofen could enhance the phosphorylation of Akt, GSK-3β and ERK1/2. (**a**–**c**) p-Akt and p-GSK-3β were not significantly changed in the hippocampal CA1 region of 2VO rats, however, hypoperfusion caused a slight but significant increase in p-ERK1/2. Chronic treatment with baclofen further significantly enhanced the phosphorylation of Akt, GSK-3β and ERK1/2. There was no significant change in the expression of total Akt, GSK-3β, and ERK1/2 (n = 4 in each group). Blots shown have been cropped to fit space requirements and run under the same experimental conditions. ^*^P < 0.05 vs sham-operated rats; ^#^*P* < 0.05 and ^##^*P* < 0.01 vs 2VO rats; ^•^*P* < 0.05 and ^••^*P* < 0.01 vs sham-operated rats.

In order to further explore the possible mechanisms of association between GABA_B_ receptors and autophagy in chronic cerebral hypoperfusion, we detected the phosphorylation as well as total level of Akt, GSK-3β, and ERK1/2 in the hippocampal CA1 area with Western blot analyses. Our results showed that p-Akt and p-GSK-3β were not significantly changed in the hippocampal CA1 region of 2VO rats. However, hypoperfusion caused a slight but significant increase in p-ERK1/2. Chronic treatment with baclofen significantly enhanced the phosphorylation of Akt, GSK-3β and ERK1/2. There were no significant changes in the expression of total Akt, GSK-3β, and ERK1/2 in each group (Fig. 8a–c).

### Baclofen could attenuate 2VO-induced reduction of GABA_A_ receptor α1 subunit surface expression

**Figure 9:**
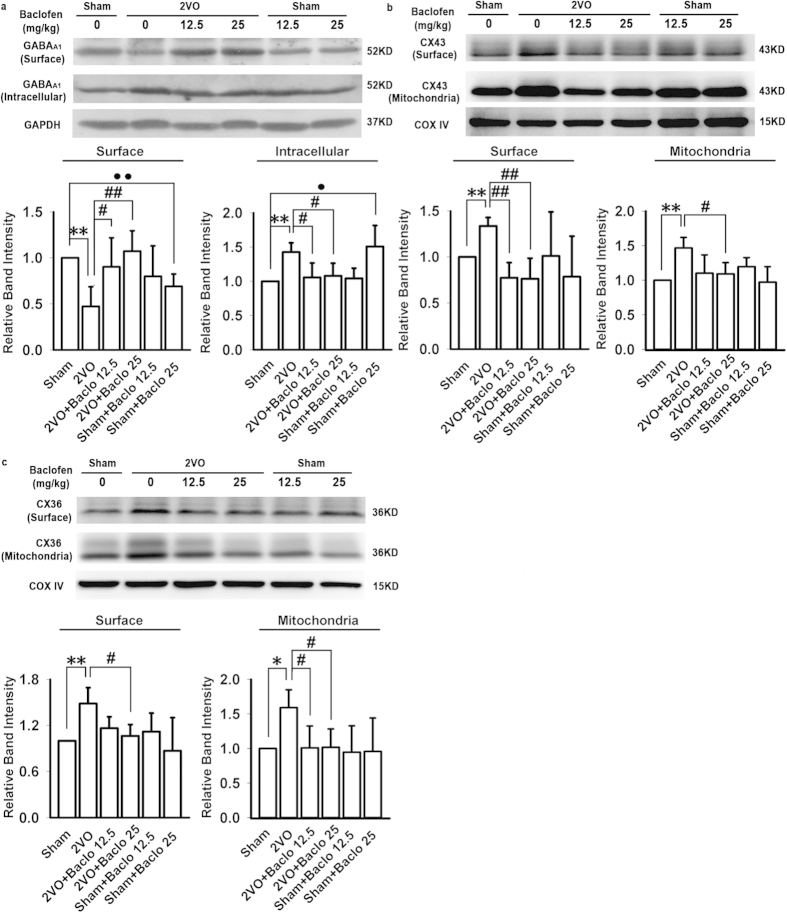
Baclofen could attenuate 2VO-induced reduction of GABA_A_ receptor α1 subunit surface expression and down-regulate the surface and mitochondrial expression of CX43 and CX36. (**a**) Five weeks after induction of hypoperfusion, the surface expression of GABA_A_ receptor α1 subunit was significantly decreased, intracellular expression of GABA_A_ receptor α1 subunit was significantly increased. Baclofen could attenuate 2VO-induced reduction of GABA_A_ receptor α1 subunit surface expression; treatment with baclofen (25 mg/kg) in sham-operated rats accelerated the decrease in the surface expression of GABA_A_ receptor α1 subunit, and the increase in the population of intracellular GABA_A_ receptor α1 subunit (n = 5 in each group). (**b**,**c**) Five weeks after induction of hypoperfusion, the surface and mitochondrial expression of CX43 and CX36 was significantly increased, baclofen could reduce CX43 and CX36 surface and mitochondrial expression in 2VO rats; treatment with baclofen in sham-operated rats did not significantly change CX43 and CX36 surface and mitochondrial expression (n = 4 in each group). Blots shown have been cropped to fit space requirements and run under the same experimental conditions. ^*^P < 0.05 and ^**^P < 0.01 vs sham-operated rats; ^#^*P* < 0.05 and ^##^*P* < 0.01 vs 2VO rats; ^•^*P* < 0.05 and ^••^*P* < 0.01 vs sham-operated rats.

In this study, we identified the surface expression of GABA_A_ receptors in the hippocampal CA1 area with Western blot analyses. No contamination with cytosolic protein was observed as GAPDH was not seen by Western blot in these samples (data not shown). Our results showed that, five weeks after induction of hypoperfusion, the surface expression of GABA_A_ receptor α1 subunit was significantly decreased, and intracellular expression of GABA_A_ receptor α1 subunit was significantly increased. Baclofen could attenuate 2VO-induced reduction of GABA_A_ receptor α1 subunit surface expression (Fig. 9a). Besides, treatment with baclofen (25 mg/kg) in sham-operated rats accelerated the decrease in the population of surface GABA_A_ receptor α1 subunit, and the increase in the population of intracellular GABA_A_ receptor α1 subunit (Fig. 9a).

### Baclofen could down-regulate the surface and mitochondrial expression of CX43 and CX36 under chronic cerebral hypoperfusion

Our results showed that, five weeks after induction of hypoperfusion, the surface and mitochondrial expression of CX43 and CX36 were significantly increased. Baclofen could reduce the surface and mitochondrial expression of CX43 and CX36 in 2VO rats; treatment with baclofen in sham-operated rats did not significantly change CX43 and CX36 surface and mitochondrial expression (Fig. 8b,c). Immunoblotting of membrane protein extracts demonstrated no reactivity with anti-GAPDH antibodies, excluding the possibility of cross-contamination by cytoplasmic fractions (data not shown).

## Discussion

In the present study, we demonstrated for the first time that chronic treatment with baclofen markedly diminished hippocampal atrophy and neuronal apoptosis in hippocampal CA1 area via the regulation of autophagy in chronic cerebral hypoperfusion in rats.

Autophagy in the CNS is a double-edged sword. Proper course of autophagy in the CNS determines the maintenance of cellular homeostasis, providing cytoprotection against stress-induced apoptosis. However, extensive autophagy destroys large proportions of the cytosol and organelles that, beyond a certain threshold, would cause irreversible cellular atrophy and trigger either apoptosis or necrotic cell death^53^. Many studies have reported that uncontrolled excessive induction of autophagy in response to ischemia injury may contribute to “autophagic cell death”, which is introduced to describe a form of programmed cell death morphologically distinct from apoptosis and characterized by the presence of intense autophagy^54^,^55^, and the inhibition of excessive autophagy can attenuate cerebral ischemia-associated neuronal damage^11^,^12^,^13^,^14^,^15^,^16^. Consistent with these studies, our present study found that baclofen could attenuate 2VO-induced increase in autophagy in hippocampal CA1 area. There is now mounting evidence that autophagy and apoptosis may share common molecular inducers and regulatory mechanism. It has been shown that Atg5 enhances caspase-dependent death though interacting directly with FADD (Fas-associated via death domain)^53^. Besides, calpain-mediated cleavage of Atg5 promotes cytochrome *c* release and caspase activation and thus switches autophagy to apoptosis^56^. Furthermore, studies have shown that increased Beclin 1 expression colocalizes with activated caspase-3 after adult focal cerebral ischemia and hypoxia-ischemia^9^,^57^, and binding of the antiapoptotic protein Bcl-2 to Beclin 1 inhibits autophagy^16^,^53^. A recent study has reported that Bcl-2 negatively regulates autophagy by inhibiting Bax and Bcl-2 homologous antagonist/killer (Bak)^58^. Our current results revealed that, under chronic cerebral hypoperfusion, baclofen could simultaneously increase the expression of Bcl-2 and Bax (especially for Bcl-2) by promoting the phosphorylation of ERK (as described below)^59^,^60^, but up-regulate Bcl-2/Bax ratio, which might both inhibit autophagy and down-regulate cleaved caspase-3^53^,^61^,^62^. Besides, it has been reported that activation of Akt does not alter the levels of Bax and Bcl-2, but Akt phosphorylation can prevent Bax translocation to mitochondria, which inhibits cytochrome *c* release^63^,^64^ and may repress autophagy^65^. All of the evidence above suggested that neuroprotection of GABA_B_ receptors activation might be closely related to its role in the regulation of autophagy. It has been reported that neurons can regulate the two opposite downstream effects of autophagy, survival and death, after ischemia^2^. It is very important to investigate the possible mechanism for this.

Studies have reported that activation of GABA_B_ receptors can enhance the phosphorylation of ERK1/2^66^, Akt and GSK-3^67^ in hippocampal neurons. Recent studies have shown that increasing ERK and Akt phosphorylation plays a critical role in mediating the neuroprotective effects under cerebral ischemia^68^,^69^,^70^,^71^,^72^. ERK and Akt not only play an important role in regulating apoptosis but also have been implicated in autophagy^16^,^73^. It means that the effects of ERK and Akt activation on the regulation of autophagy may be at least partly involved in ERK/Akt-mediated neuroprotection.

It has been reported that activation of MEK/ERK downstream of AMPK leads to disassembly of mTORC1 and mTORC2, and an increase in Beclin 1 expression^7^. In this report, our results showed that, under chronic cerebral hypoperfusion, treatment with baclofen significantly enhanced the phosphorylation of ERK1/2, but inhibited the expression of Beclin 1. The main reasons for this inconsistent result may be as follows. On the one hand, under chronic cerebral hypoperfusion, baclofen-induced ERK1/2 phosphorylation can increase Beclin 1 expression moderately. On the other hand, baclofen-induced Akt phosphorylation may significantly down-regulate the expression of Beclin 1, since it has been reported that the PI3K/Akt inhibitor LY294002 abrogates the down-regulation effect of melatonin on Beclin-1 expression in rat model of transient focal cerebral ischemia^74^. The combined effect of these was that activation of GABA_B_ receptors reduced Beclin 1 expression and inhibited autophagy under chronic cerebral hypoperfusion. However, treatment with baclofen in sham-operated rats did not significantly change the expression of Beclin 1 compared with sham operated rats. It meant that, under physiological conditions, baclofen-induced ERK1/2 and Akt phosphorylation could keep autophagy in balance. One study has shown that transiently or moderately activated MEK/ERK leads to the inhibition of either mTORC1 or mTORC2 and the moderate increase in Beclin 1 expression, resulting in cytoprotective autophagy^7^. Wang *et al.* have reported that activation of ERK induces protective autophagy against the injury of transient middle cerebral artery occlusion (MCAO) followed by reperfusion^75^. It meant that baclofen-induced ERK1/2 phosphorylation might accelerate cytoprotective autophagy by increasing Beclin 1 expression moderately. Besides, activation of ERK can up-regulate the expression of Bcl-2^60^, which may similarly suppress cytodestructive autophagy, since Bcl-2 is associated with Beclin1^16^ and Bax^58^.

Many studies have shown that the activated Akt kinase not only suppresses the proapoptotic function of Bax^63^,^64^,^76^ but also inhibits the activity of hamartin (TSC1) and tuberin (TSC2) protein complex, which reduces the GTPase activity of Ras homolog enriched in brain (Rheb) and leads to activation of mTOR and subsequent inhibition of autophagy^16^. In this report, we found that the activity of Akt was much higher in baclofen-treated rats than that in 2VO and sham-operated rats. We then examined the changes in GSK-3β activity, because Akt was an upstream regulator of GSK-3β phosphorylation which had also been proposed as an intracellular signalling mechanism mediating autophagy. It has been reported that phosphorylation of GSK-3β can be conducive to autophagy repression^52^. Our data revealed that, under chronic cerebral hypoperfusion, baclofen significantly enhanced GSK-3β phosphorylation, which could both increase p-mTOR levels and reduce the expression and function of Beclin 1^77^. A recent study has shown that the inhibition of autophagy via activation of PI3K/Akt pathway has neuroprotective role in transient global ischemia^17^. Together, our observations indicated that baclofen might suppress cytodestructive autophagic activity through Akt-GSK-3β-p-mTOR-Beclin 1 signaling pathway under chronic cerebral hypoperfusion.

In addition, a more recent study has reported that plasma membrane Cx43 and other members of the Cx family contribute to negatively modulate autophagy, which seems independent of their function in intercellular communication and signaling, but requires the physical interaction of autophagy precursors, such as Atg16^52^. Studies have reported that CX43 and CX36 are significantly increased in animal models of unilateral middle cerebral artery occlusion (MCAO), oxygen-glucose deprivation (OGD)^50^, and transient brain ischemia^44^,^51^,^78^. Inhibiting the expression and function of CX43 and CX36 may be involved in the neuroprotection^44^,^50^. Thus, we speculated that, the increased expression of CX43 and CX36 could reduce cytoprotective autophagy and promote neuronal death during ischemia. In the present study, our results showed that, five weeks after induction of hypoperfusion, the surface expression of CX43 and CX36 was significantly increased, and baclofen could reduce CX43 and CX36 surface expression. It meant that, during chronic cerebral hypoperfusion, the up-regulation of CX43 and CX36 surface expression suppressed cytoprotective autophagy, and mediated the spread of pro-death signals that resulted in widespread neuronal demise. Baclofen-induced suppressed the increase in CX43 and CX36 surface expressions, thus helping neurons survive.

Studies have reported that GABA_A_ receptors activation can down-regulate the expression of Cx43^43^ and Cx36^44^ in the CNS. In the present study, our results revealed that chronic GABA_B_ receptors agonist exposure could attenuate 2VO-induced reduction of the surface expression of GABA_A_ receptors α1 subunit. However, treatment with baclofen (25 mg/kg) in sham-operated rats accelerated the decrease in the population of surface GABA_A_ receptor α1 subunit, which was consistent with previous studies that had shown activation of GABA_B_ receptors could reduce GABA_A_ receptors -mediated currents under normal conditions^79^,^80^. The main reasons why activation of GABA_B_ receptors played different roles in GABA_A_ receptors expression under normal condition and chronic cerebral hypoperfusion may be as follows. A recent study has reported that an increase in the intracellular Ca^2+^ concentration can enhance the desensitization of GABA_A_ receptors in the barrel cortex in PRIP-1/2 double-knockout (PRIP-DKO) mice^81^. So we speculated that chronic hypoperfusion-induced increase in the intracellular Ca^2+^ concentration could enhance the desensitization of GABA_A_ receptors resulting in the reduction of GABA_A_ receptors surface expression. Previous studies have reported that dose-dependent administration of baclofen depresses Ca^2+^-influx^82^,^83^,^84^. Besides, it has been demonstrated that GABA_B_ receptors activation suppresses NMDA receptors - mediated Ca^2+^ influx by attenuating the activity of Src in rat four-vessel occlusion (4-VO) ischemic model^31^. Thus, under chronic cerebral hypoperfusion, activation of GABA_B_ receptors might contribute to the resensitization of GABA_A_ receptors and restore GABA_A_ receptors surface expression by depressing Ca^2+^-influx. However, under normal conditions, in order to maintain the balance of excitation and inhibition, the inhibiting effect of GABA_B_ receptors activation on neuronal excitability by depressing Ca^2+^-influx may be attenuated by down-regulating GABA_A_ receptors surface expression. Together, our results demonstrated that baclofen-induced inhibition of CX43 and CX36 surface expression might promote cytoprotective autophagy by improving GABA_A_ receptor α1 subunit surface expression.

Furthermore, mitophagy plays a protective effect during cerebral ischemia^85^,^86^. A significant increase in CX43 and CX36 mitochondrial expression during chronic cerebral hypoperfusion might inhibit mitophagy, impede mitophagy-related mitochondrial clearance, and then aggravate ischemia-induced neuronal cell death. Baclofen-induced inhibition of CX43 and CX36 mitochondrial expression might promote mitophagy, and then inhibit downstream apoptosis. Further investigations are underway in our laboratory.

**Figure 10:**
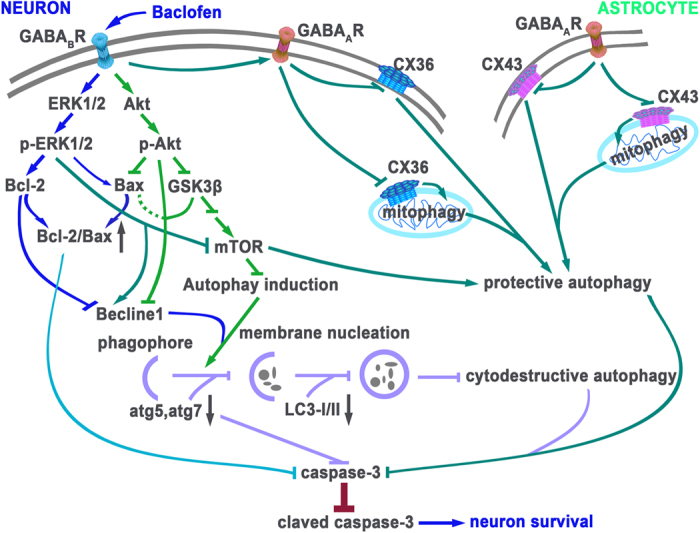
A mechanistic explanation for bi-directional regulative effects of baclofen on autophagy in rat hippocampal CA1 area under chronic cerebral hypoperfusion. Activation of GABA_B_ receptors could regulate autophagy activity via possible mechanisms as follows: on the one hand, activation of Akt both enhanced GSK-3β phosphorylation, leading to activation of mTOR, and significantly down-regulated the expression of Beclin 1, subsequently inhibited cytodestructive autophagy; Akt phosphorylation could prevent Bax translocation to mitochondria, which both inhibited cytochrome *c* release and might repress cytodestructive autophagy, which also attenuated chronic hypoperfusion-induced neuronal damage. Activation of ERK could up-regulate the expression of Bcl-2, which negatively regulate cytodestructive autophagy through association with Beclin1 and Bax; activation of GABA_B_ receptors could attenuate 2VO-induced increase in the expression of atg5 and atg7, which also inhibited cytodestructive autophagy and neuronal apoptosis; on the other hand, baclofen-induced ERK1/2 phosphorylation might accelerate cytoprotective autophagy by increasing Beclin 1 expression moderately; activation of GABA_B_ receptors improved GABA_A_ receptor α1 subunit surface expression, resulting in the down-regulation of CX43 (astrocyte) and CX36 (neuron) surface and mitochondrial expression, and then enhanced cytoprotective autophagy. Together, bi-directional regulative effects of GABA_B_ receptors activation on autophagy reversed neuronal damage and cognitive impairment induced by chronic cerebral hypoperfusion. The figure was drawn by Li Liu and Chang-jun Li.

In conclusion, our present results demonstrated that, under chronic cerebral hypoperfusion, activation of GABA_B_ receptors suppressed cytodestructive autophagic activity through Akt/ERK-Bcl2-Beclin1 signaling pathway, while up-regulated protective autophagy through the activation of GABA_A_ receptor-CX43/CX36 signaling pathway. The bi-directional regulative effects of GABA_B_ receptors activation on autophagy reversed neuronal damage induced by 2VO (Fig. 10).

## Materials and Methods

### Animals

All experiments were performed following an institutionally approved protocol in accordance with relevant guidelines and regulations of the Review Committee for the Care and Use of Laboratory Animals of Tongji Medical College, Huazhong University of Science and Technology. Adult male Sprague-Dawley rats of clean grade (approval number: SCXK(E)2010-0007, No. 00008580), aged 2–3 months, weighing 220–250 g, were provided from Experimental Animal Center, Tongji Medical College, Huazhong University of Science and Technology. Animals were group-housed with free access to water and food with a 12 h light/dark cycle and a thermoregulated environment, and adapted to these conditions for at least 7 days before experiments. All efforts were made to minimize both the suffering and to reduce number of animals used.

### OGD-Rep injury of brain slices and drug treatment

The rats were anesthetized with chloral hydrate (350 mg/kg, intraperitoneal injection, i.p.). The cerebrum was removed rapidly and placed in ice-cold artificial cerebrospinal fluid (aCSF) bubbled with 95% O_2_ and 5% CO_2_. The aCSF containing (mM): NaCl 126, KCl 3.5, NaH_2_PO_4_ 1.2, mgCl_2_ 1.3, CaCl_2_ 2.0, D-(+)-glucose 11, NaHCO_3_ 25; 290 mosm, gassed with 95% O_2_ and 5% CO_2_ (pH7.4). The cerebrum was immediately sectioned with a Mcllwain tissue chopper (The Mickle Laboratory Engineering Co. LTD, USA) into 400 μm coronal slices in ice-cold aCSF bubbled with 95% O_2_ and 5% CO_2_. The slices were placed on top of the semiporous membranes in six well trays. All the slices were maintained in the plating medium contained DMEM-F/12, 10% fetal bovine serum (FBS; Gibco, Grand Island, NY, US), penicillin and streptomycin (100 U/ml) at 37 °C for 30 min recovery. For OGD, the medium was replaced with sugar-free DMEM, gassed with 95% N_2_ and 5% CO_2_ at 37 °C for 30 min. For reperfusion, the slices were refreshed with normal culture medium for 6 h. Baclofen (100 μM)^37^ was dissolved in normal culture medium and added to the slices at the onset of reperfusion.

### Animal model of chronic cerebral hypoperfusion and treatment schedules

Model preparation was described in detail in our previous study^87^. Two weeks after chronic cerebral hypoperfusion, repeated drug treatment of all groups was performed once daily at 20:00 p.m.–21:00 p.m. during the last 21 days. Baclofen (Meryer Chemical Technology Co., Ltd, Shanghai, China) was dissolved in saline at concentration of 1.25 mg/ml and 2.5 mg/ml^31^,^35^,^36^. All groups were treated with baclofen in a volume of 10 ml/kg or same volume of normal saline (NS) by i.p. injection. Five weeks after 2VO, rats were killed by decapitation under anesthesia and carried out biochemical studies as described below.

### Immunofluorescence and Hematoxylin and Eosin (H&E) staining

After the successive perfusion of rats by intracardiac injection of 0.9% saline solution and 4% paraformaldehyde (PFA) in 0.1 M phosphate buffer (PB), brains were harvested and postfixed in 4% PFA overnight. After conventional paraffin embedding and serial section (5 μm), immunohistochemistry staining was sequentially performed following incubation in anti-LC3 (1: 500, PM036, MBL) overnight at 4 °C and Fluorescein (FITC)-conjugated Affinipure Donkey Anti-Rabbit IgG(H+L) (SA00003-8, Proteintech Group Inc, China) for 2 h, and imaged with Olympus FluoView 1200 confocal microscope system (Olympus Corporation, Japan). A quantitative analysis of LC3 staining was performed using analySIS software (analySIS 3.0; Soft Imaging System)^88^. TUNEL assay uses the *In Situ* Cell Death Detection Kit (11684817910, Roche, Basel, Switzerland) according to the manufacturer’s protocol. Briefly, the paraffin sections were dewaxed and rehydrated, followed by incubation with protease K (20 mg/ml) for 30 min at 37 °C and the TUNEL reaction mixture 2 h at 37 °C. The nuclei were counterstained with DAPI. Numbers of total nuclei and TUNEL-positive nuclei were counted and the apoptosis ratio was calculated as follows: apoptosis ratio = (number of TUNEL-positive nuclei/number of total nuclei) × 100%. Hematoxylin and eosin staining was performed as follows: hematoxylin staining for 15 min, hydrochloric acid alcohol solution for 35 s decoloring, eosin staining for 10 minutes and 90% ethanol for 40 s decoloring. Then neutral balsam was used for mounting and the section was observed and photographed under the microscope.

### Western blotting analysis

Rats were decapitated under anesthesia. Brains were rapidly removed and placed into ice-cold artificial cerebrospinal fluid (ACSF, saturated with 95% O_2_/5% CO_2_), and were cut into coronal slices (400 μm) and then cornu ammonis 1 (CA1) region was separated carefully from the hippocampus under a dissecting microscope^89^. Samples were stored at −80 °C until ready for use. Membrane and mitochondrial protein extracts were prepared using ProteoExtract Native Membrane Protein Extraction Kit (71772-3, Calbiochem/Merck Biosciences) and Mitochondria Isolation Kit (AR0156, Boster, Wuhan, China) respectively. Protein concentration was determined using a BCA Protein Assay Kit (Pierce). Equal amounts of protein samples (80 μg) were separated by 10% or 15% SDS/PAGE gel electrophoresis and then transferred to Polyvinylidene difluoride (PVDF) membranes (03010040001, Roche). After blocking with 5% non-fat milk powder in Tris-buffered saline containing 0.1% Tween-20 (TBST) for 1 h at room temperature, transferred membranes were incubated overnight at 4 °C with primary antibodies to anti-GABA_A_ receptor alpha 1 (1: 1000, ab33299, abcam), anti-LC3 (1: 1000, PM036, MBL), Anti-mTOR (1: 1000, 2983, Cell Signaling), anti-phospho- mTOR (Ser9)(1: 1000, 5536, Cell Signaling), anti-atg5 (1: 500, NB110-53818, Novus), anti-atg7 (1: 200, ab53255, abcam), anti-Beclin1 (1: 1000, NB500-249, Novus), Anti-Akt (1: 1000, 9272, Cell Signaling), anti-phospho-Akt (Ser473) (1: 1000, 4060, Cell Signaling), Anti-p44/42 MAPK (Erk1/2) (1: 1000, 9102, Cell Signaling), anti-phospho-p44/42 MAPK (Erk1/2) (Thr202/Tyr204) (20G11)(1: 1000, 4376, Cell Signaling), Anti-GSK-3β (1: 1000, 9315, Cell Signaling), anti-phospho-GSK-3β (Ser9)(1: 1000, 9336, Cell Signaling), anti-caspase-3 (1: 1000, 9662, Cell Signaling), anti-cleaved caspase-3 (Asp175) (1: 1000, 9664, Cell Signaling), anti-Bax (1: 1000, 2772, Cell Signaling), anti-Bcl2 (1: 1000, AF0769, Affinity Bioscience), anti-Connexin43 (1: 1000, 71-0700, Invitrogen), anti-Connexin36 (1: 300, 51-6300, Invitrogen), anti-COX IV (1: 5000, A01060, Abbkine), anti-GAPDH (1: 5000, cw0100, Cwbiotech) or anti-alpha tubulin (1: 5000, ab125267, abcam). The antigen-antibody complexes were visualized with goat anti-rabbit or goat anti-mouse horseradish peroxidase (HRP) conjugated secondary antibodies (1: 5000; Proteintech Group Inc, China) by using immobilon Western chemiluminescent HRP substrate (WBKLS0500, Millipore). The optical density of bands was measured using NIH Image J software, and results were normalized to GAPDH, COX IV or alpha tubulin in each sample lane. All assays were performed at least four times.

### Statistical Analysis

All analyses were performed using SPSS 16.0 software (SPSS Inc., USA) and data were presented as mean ± SD. Differences between groups were evaluated using one- or two -way analysis of variance (ANOVA), as appropriate. The t-test was used for testing differences between two groups. *P* < 0.05 was considered statistically significant.

## Additional Information

**How to cite this article**: Liu, L. *et al.* Baclofen mediates neuroprotection on hippocampal CA1 pyramidal cells through the regulation of autophagy under chronic cerebral hypoperfusion. *Sci. Rep.* **5**, 14474; doi: 10.1038/srep14474 (2015).
